# Bladder Metastasis From Lung Adenocarcinoma: A Rare Case

**DOI:** 10.7759/cureus.83434

**Published:** 2025-05-03

**Authors:** Nicola Fazaa, Etan Eigner, Inbal Farkash, Gilad Amiel, Azik Hoffman

**Affiliations:** 1 The Ruth and Bruce Rappaport Faculty of Medicine, Technion Medicine, Haifa, ISR; 2 Urology, Rambam Health Care Campus, Haifa, ISR; 3 Pathology, Rambam Health Care Campus, Haifa, ISR

**Keywords:** bladder metastasis, gross hematuria, immunohistochemistry (ihc), pulmonary adenocarcinoma, transurethral resection of bladder tumor (turbt)

## Abstract

Bladder metastasis from primary lung adenocarcinoma is an infrequent clinical entity. Due to its rarity and nonspecific presentation, it is often misdiagnosed as primary urothelial carcinoma. We report the case of a 65-year-old male with a history of metastatic lung adenocarcinoma presenting to the emergency room with sudden gross hematuria. Imaging revealed a new bladder lesion. Following transurethral resection, histopathological and immunohistochemical analysis confirmed metastatic lung adenocarcinoma (thyroid transcription factor-1-positive/cytokeratin 7-positive/cytokeratin 20 negative/GATA binding protein 3-negative or TTF-1+/CK7+/CK20-/GATA3-). The case was discussed at the Genitourinary Oncology Tumor Board, and given the prognosis of metastatic lung adenocarcinoma, the patient opted for palliative care. This case highlights the importance of considering metastatic spread to the urinary bladder in patients with advanced malignancy who develop new urinary symptoms. Accurate diagnosis through histopathology and immunohistochemistry is essential for guiding appropriate management.

## Introduction

Lung adenocarcinoma is the most common histological subtype of lung cancer and is known for its ability to metastasize to various organs, including the liver, bones, adrenal glands, and brain [1]. Bladder metastasis from lung adenocarcinoma is exceptionally rare, with fewer than 15 cases reported over the past two decades [2]. The mechanism of metastasis to the bladder is not well understood, but hematogenous and lymphatic dissemination have been proposed as possible pathways [3]. Most bladder metastases from lung cancer present with hematuria, which is often initially mistaken for primary urothelial carcinoma, leading to diagnostic challenges [2]. In this report, we present a case of bladder metastasis from lung adenocarcinoma and discuss its clinical, histopathological, and immunohistochemical features in the context of the available literature.

## Case presentation

A 65-year-old male, with a significant smoking history (60 pack-years), was diagnosed with lung adenocarcinoma with metastases to the adrenal glands, bones, and lymph nodes. The patient underwent palliative radiation therapy to the right knee, followed by a right above-knee amputation. He was subsequently treated by systemic immunotherapy with pembrolizumab. Three months later, during a revision surgery for his amputation, the patient developed gross hematuria. A three-way catheter was placed to facilitate continuous bladder irrigation.

**Figure 1:**
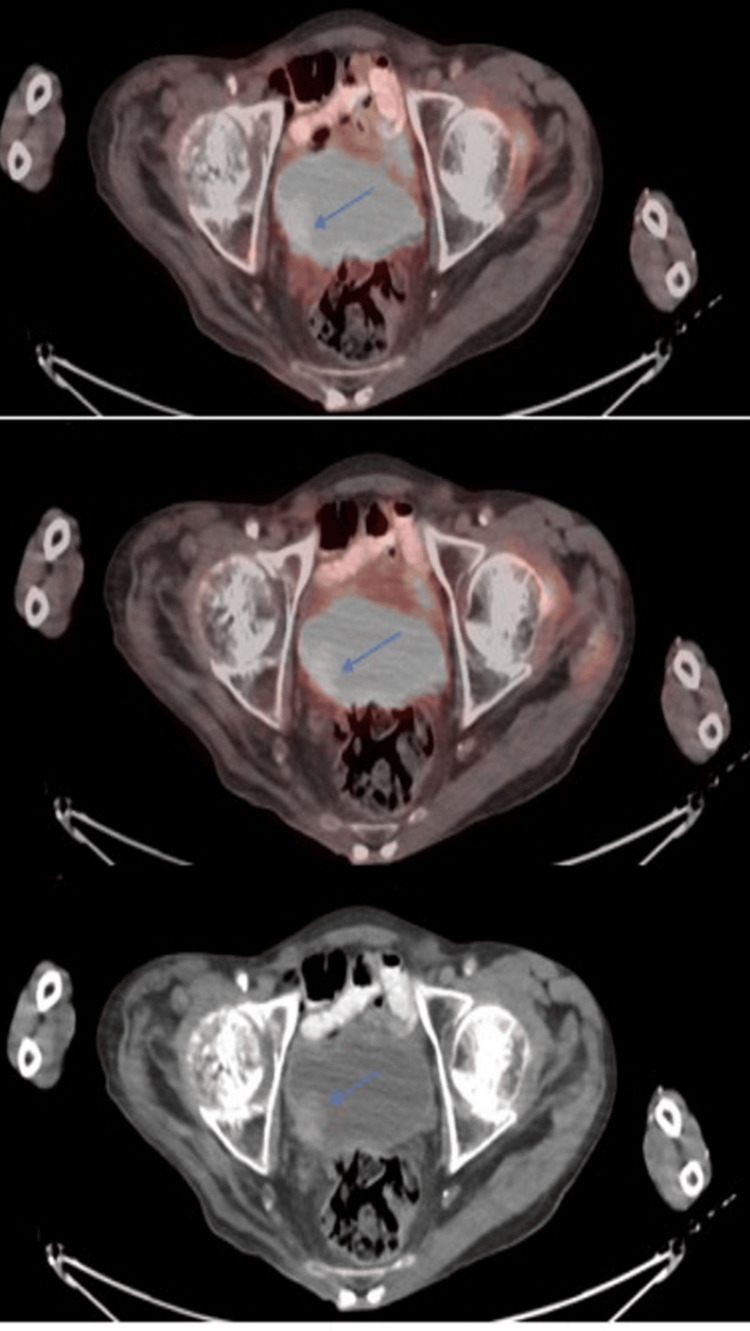
Focal hypermetabolic mass in the urinary bladder on PET-CT.

A repeat PET-CT scan one month later identified a 2-cm space-occupying lesion located on the right bladder wall (Figure 1), which had not been seen on a prior CT scan a few months ago. Four weeks later, the patient underwent transurethral resection of the bladder tumor (TURBT), during which a 3 cm solid mass was resected from the right bladder wall. Concurrently, a transurethral resection of the prostate (TURP) was performed to facilitate a trial without a catheter, which was successful.

**Figure 2:**
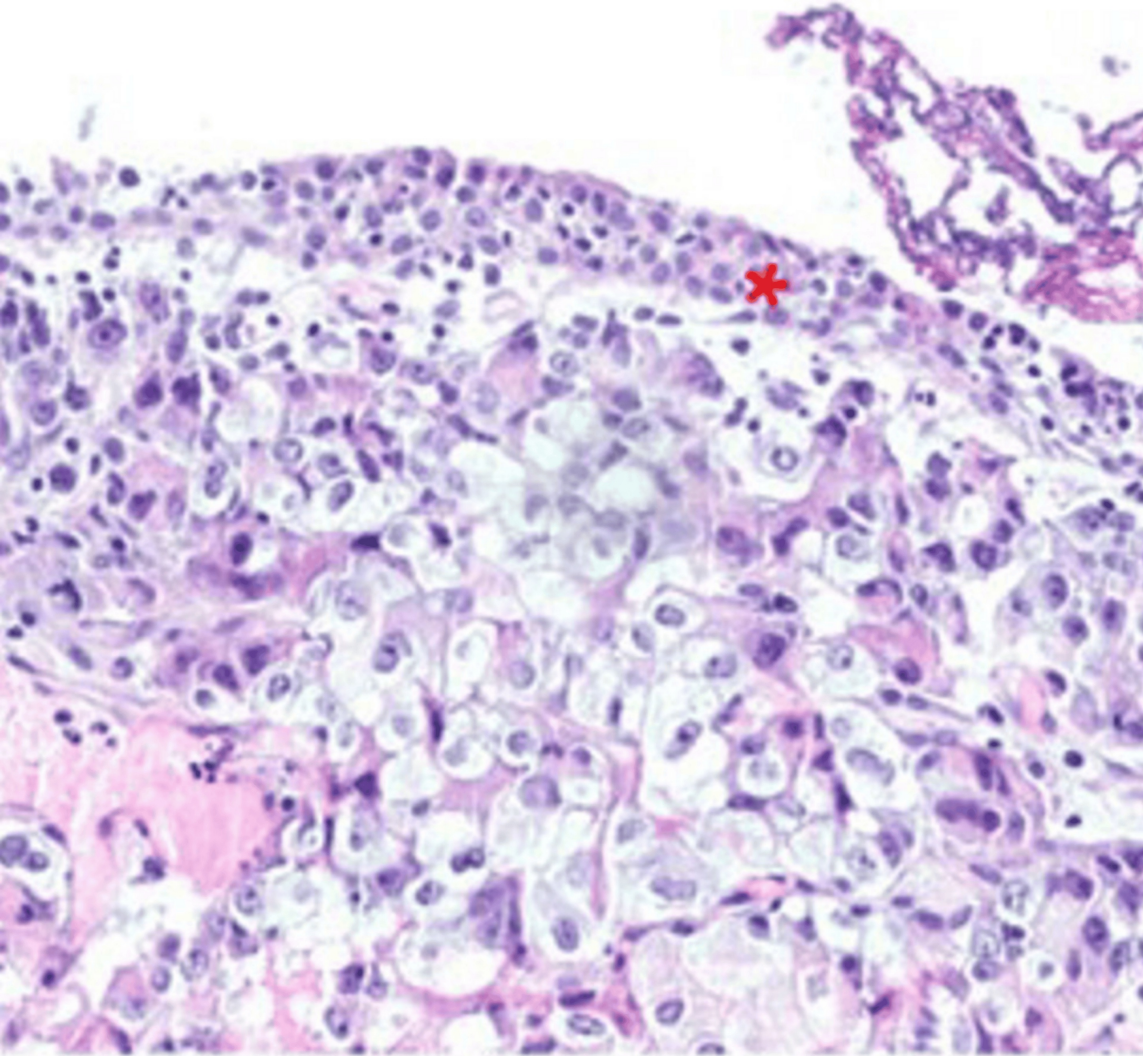
Carcinoma infiltrates the lamina propria beneath the benign urothelium.

The histopathological examination demonstrated infiltration of the urothelium, lamina propria, and muscularis propria by malignant cells. Hematoxylin and eosin (H&E) staining (Figure 2) revealed an atypical cellular infiltrate beneath the urothelial surface.

**Figure 3:**
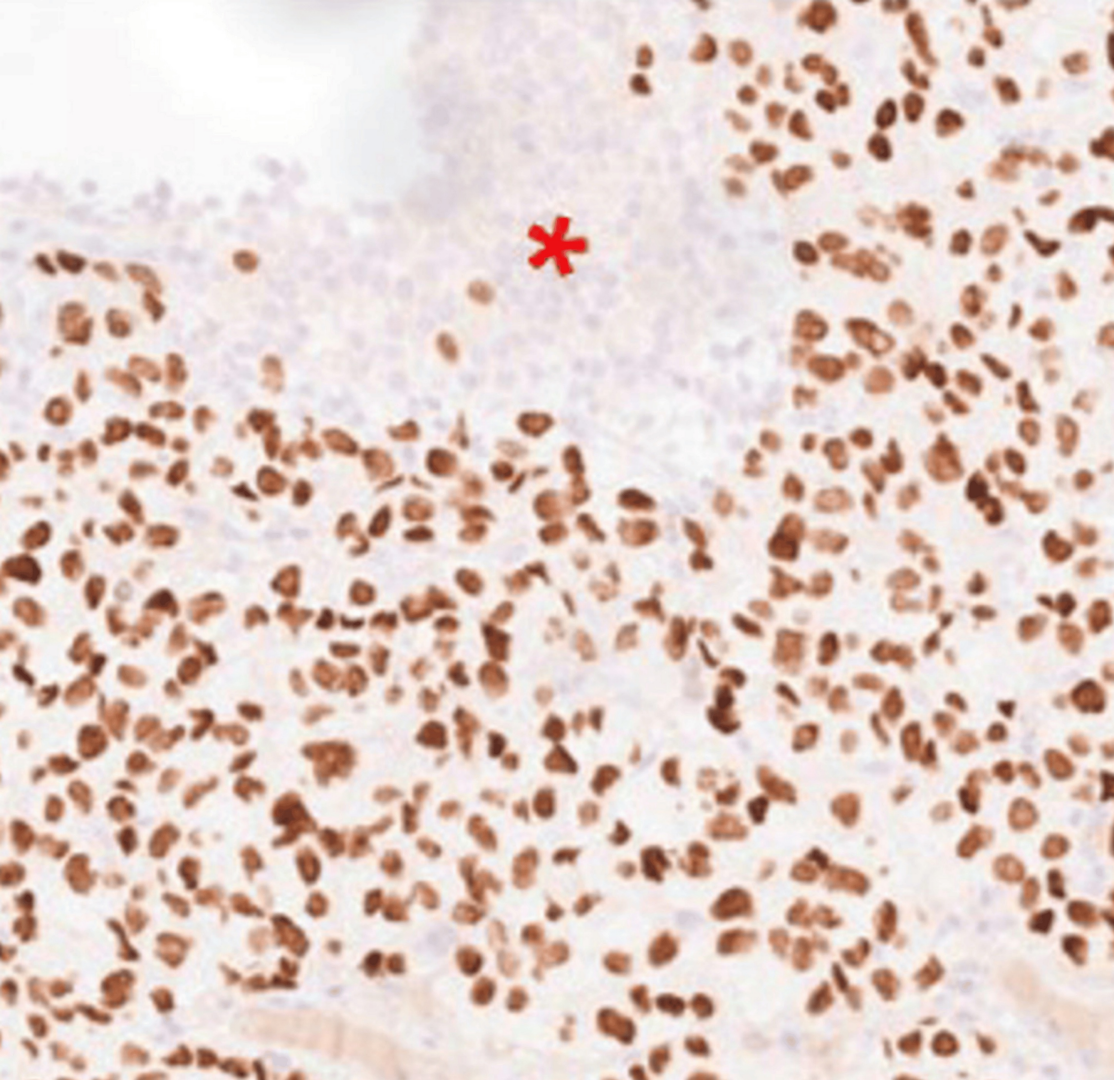
TTF-1 positive carcinoma cells in contrast with the negative benign urothelium.

**Figure 4:**
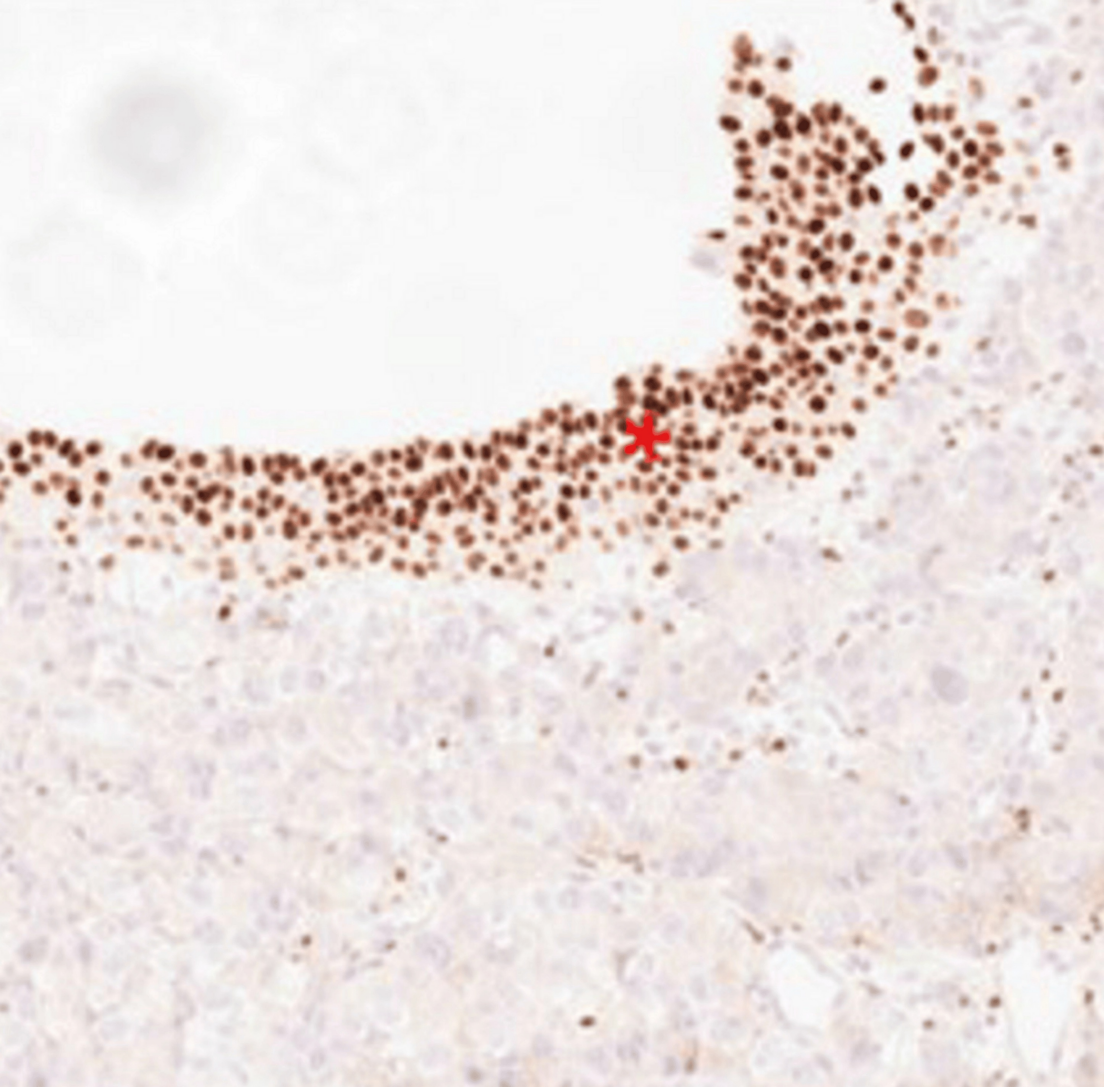
GATA3 positive in the benign urothelium. The carcinoma cells are negative for GATA3.

Immunohistochemical analysis showed strong nuclear uptake for thyroid transcription factor-1 (TTF-1) (Figure 3) and positivity for cytokeratin 7 (CK7), while cytokeratin 20 (CK20) and GATA3 staining (Figure 4) were negative. This immunophenotypic profile (TTF-1+/CK7+/CK20-/GATA3-) is inconsistent with primary urothelial carcinoma and instead strongly supports a diagnosis of metastatic lung adenocarcinoma involving the bladder wall.

Following the confirmation of metastatic lung adenocarcinoma involving the bladder based on histopathological and immunohistochemical findings, the case was presented to the multidisciplinary Genitourinary Oncology Tumor Board. Given the patient's overall disease burden, as seen in whole-body imaging, and the prognosis associated with metastatic lung adenocarcinoma, he was referred back to his primary oncologist to discuss potential treatment options. After a comprehensive review of therapeutic strategies, including systemic therapy, the patient opted for palliative care, prioritizing symptom management and quality of life.

## Discussion

Bladder metastases from lung adenocarcinoma are exceptionally rare, with fewer than 15 cases documented over the past two decades [2]. Patients typically present with hematuria, which can easily be misinterpreted as indicative of primary urothelial carcinoma [4], especially since smoking is a common risk factor for both bladder and lung carcinomas. This underscores the imperative for meticulous histopathological and immunohistochemical evaluations to achieve accurate diagnosis.

Metastases from distant primary tumors represent less than 2% of bladder cancers. Primary tumor locations with bladder metastases described in the literature are stomach, melanoma, breast, and lung [5,6]. The appearance of these tumors on cystoscopy could be variable and sometimes nonspecific and may look similar to other primary and secondary bladder tumors [3]. Table 1 provides a comprehensive overview of reported cases of lung adenocarcinoma presenting as a metastatic lesion in the bladder.

**Table 1:** Pulmonary adenocarcinoma metastasis to the bladder – clinical and pathological features.

Age	Sex	Clinical presentation	Findings from cystoscopy	Immunohistochemistry and genetic marker results	Reference
52	M	Microscopic hematuria, dysuria	Tumor located at the apex of the bladder	CK-7(+), TTF-1(+), CK-20(-)	[9]
61	M	Hydronephrosis	Thickened bladder wall	EGFR19(+), T790M(-)	[10]
63	F	Arthralgia	Solid lesion measuring 3cm on the left side of the bladder wall	CK7(+), S100P(+), CK20(-), GATA3(+), CDX-2(-), TTF-1(-), NapsinA(-)	[8]
81	F	Abdominal pain	Normal mucosal appearance with external compression	TTF-1(-), CK7(+), NapsinA(+)	[11]
83	M	Gross hematuria	Bladder diverticulum observed in the right posterior wall	CK20(-), CK7(+), TTF-1(+), PSA(-), AR(-)	[12]
55	M	Lower urinary tract symptoms (LUTS)	Involvement of the trigone and right lateral bladder wall	CK7(+), TTF1(+), CK20(-), PSA(-)	[5]
78	M	Gross hematuria	3mm papillary tumor, right lateral wall	TTF-1(+), CK-7(+), CK-20(-)	[13]
65	F	Gross hematuria	Numerous solid lesions within the bladder	TTF-1(+), CK7(+), CK20(-), CD15(-)	[6]
53	M	Hematuria	Normal mucosal lining with invasion of the detrusor muscle	TTF-1(+), CK-7(+), CK-20(-)	[14]
71	M	Hematuria	Involvement along the urinary tract without muscle invasion	CK-7(+), TTF-1(+), NapsinA(+), CK20(-), PSA(-), P504S(-)	[15]
86	F	LUTS	Nodular lesion with calcified areas in the left bladder wall	TTF-1(+), CK7(+), CK20(-), napsinA(-), p53(+), GATA3(+), Uroplakin III(-)	[16]
40	M	Hematuria	Not specified	TTF-1(+), CK7(+), CK20(+)	[17]
72	F	Gross hematuria	Space-occupying lesions	CK7(+), TTF-1(+), NapsinA(+), CK20(+), GATA3(-), CR(-), CD163(-), PAX-8(-).	[2]

Immunohistochemistry plays a pivotal role in differentiating primary bladder tumors from metastatic lesions. Markers such as TTF-1 and CK7 are commonly expressed in lung adenocarcinomas, whereas CK20 and GATA3 are typically negative [5,7]. This immune profile aids in distinguishing metastatic lung adenocarcinoma from primary bladder adenocarcinoma and other malignancies [8].

The prognosis for patients with bladder metastasis from lung adenocarcinoma is generally poor, reflecting the aggressive nature of the primary disease and most often the presence of widespread metastases. Treatment strategies are primarily palliative, focusing on systemic chemotherapy tailored to the primary lung cancer. Localized treatments, such as transurethral resection or radiation therapy, may be offered to alleviate symptoms such as hematuria, but do not significantly improve overall survival [2,5].

A comprehensive review of the literature reveals that bladder metastases from lung adenocarcinoma often occur in the context of disseminated disease. For instance, Liu et al. described a 55-year-old patient with lung adenocarcinoma who developed bladder metastasis, highlighting the aggressive progression of the disease [2]. Similarly, other studies have documented cases in which bladder metastasis was detected as part of a widespread metastatic spread, further emphasizing the need for systemic therapeutic approaches [4,5,7].

This case contributes to the limited body of literature on this rare phenomenon, emphasizing the necessity to highlight this possible spread pattern, improve clinical awareness, and prevent misdiagnosis. Given that lung cancer remains a leading cause of cancer-related mortality, the possibility of atypical metastatic patterns should always be considered in patients with unusual presentations.

## Conclusions

This case highlights the importance of considering metastatic disease in patients with a known primary malignancy who develop new urinary symptoms, such as hematuria. Accurate diagnosis through histopathological and immunohistochemical analysis is essential to guide proper management and avoid unnecessary interventions.
